# Structural Changes Induced by Acupuncture in the Recovering Brain after Ischemic Stroke

**DOI:** 10.1155/2018/5179689

**Published:** 2018-05-23

**Authors:** Ping Wu, Yu-mei Zhou, Chen-xi Liao, Yu-zhi Tang, Yong-xin Li, Li-hua Qiu, Wei Qin, Fang Zeng, Fan-rong Liang

**Affiliations:** ^1^Acupuncture and Tuina School/Third Teaching Hospital, Chengdu University of Traditional Chinese Medicine, Chengdu, Sichuan Province, China; ^2^Institute of Clinical Anatomy, School of Basic Medical Sciences, Southern Medical University, Guangzhou, Guangdong Province, China; ^3^Radiology Department, West China Hospital of Sichuan University, Chengdu, Sichuan Province, China; ^4^Life Sciences Research Center, School of Life Sciences and Technology, Xidian University, Xi'an, Shaanxi Province, China

## Abstract

The aim of this study was to observe the grey matter (GM) tissue changes of ischemic stroke patients, to explore the therapy responses and possible mechanism of acupuncture. 21 stroke patients were randomly assigned to receive either acupuncture plus conventional (Group A) or only conventional (Group B) treatments for 4 weeks. All patients in both groups accepted resting-state functional magnetic resonance (fMRI) scan before and after treatment, and the voxel-based morphometry (VBM) analysis was performed to detect the cerebral grey structure changes. The modified Barthel index (MBI) was used to evaluate the therapeutic effect. Compared with the patients in Group B, the patients in Group A exhibited a more significant enhancement of the changes degree of MBI from pre- to post-treatment intervention. VBM analyses found that after treatment the patients in Group A showed extensive changes in GMV. In Group A, the left frontal lobe, precentral gyrus, superior parietal gyrus, anterior cingulate cortex, and middle temporal gyrus significantly increased, and the right frontal gyrus, inferior parietal gyrus, and middle cingulate cortex decreased (*P* < 0.05, corrected). In addition, left anterior cingulate cortex and left middle temporal gyrus are positively related to the increase in MBI score (*P* < 0.05, corrected). In Group B, right precentral gyrus and right inferior frontal gyrus increased (*P* < 0.05, corrected). In conclusion, acupuncture can evoke pronounced structural reorganization in the frontal areas and the network of DMN areas, which may be the potential therapy target and the potential mechanism where acupuncture improved the motor and cognition recovery.

## 1. Introduction

Stroke is the second major cause of death in the world [1] and the first leading cause of adult disability. It is reported that there are 1.5 to 2 million new strokes per year in China [2]. The symptoms caused by stroke, such as hemiplegia, cognitive disorder, aphasia, and dysphagia, greatly affect the ability of patients to perform activities of daily living (ADL), as well as social participation, imposing a great burden on families and communities in many developed countries [3]. Stroke became an important public health-care and social issue because of its high prevalence, unsatisfactory treatment options, large medical burden, and serious reduction in quality of life (QQL). Hence, both patients and practitioners desire effective alternative therapies.

Acupuncture, a major medical resource, has been extensively used to treat stroke for several millennia. Acupuncture is regarded as a very effective therapeutic intervention and is becoming more and more popular in western countries [4, 5]. During the past decades, a considerable number of clinical and experimental studies have shown its safety and potential beneficial effects in the poststroke rehabilitation [6]. The latest review in 2016 has indicated that acupuncture was demonstrated to be a promising tool for improving functional recovery in stroke patients [7]. However, we still have not been able to explore its exact so far.

In recent years, some studies suggested that structural neuroplastic changes in the brain, such as grey matter volume, might be closely related to both behavioral recovery and active rehabilitation after stroke [8–10]. Miao et al. [11] found that well-recovered stroke patients exhibited significantly increased grey matter volume (GMV) in contralesional supplementary motor area (SMA). In another motor rehabilitation therapy study, stroke patients have shown a high GMV in frontal and parietal sensory-motor areas and in the hippocampus [12]. Moreover, our previous research [13] also found that stroke patients showed some changes in cerebral GMV, including precentral gyrus, cerebellum, and middle frontal gyrus compared with healthy subjects. However, whether acupuncture can induce the structural changes was not addressed in the previous study. Hence, based on the converging evidence, we hypothesize that acupuncture therapy will change the GMV of the key regions by inducing the structure neuroplastic changes in the cerebral cortex and subcortex. Hence, the present study aimed to investigate the influence of acupuncture on cerebral grey matter volume in order to explore the potential central mechanism of acupuncture treatment.

## 2. Methods

### 2.1. Participants

In this study, we recruited the ischemic stroke patients from the 1st Teaching Hospital of Chengdu University of Traditional Chinese Medicine (CDUTCM). All the subjects need to meet the following criteria: (1) were diagnosed with ischemic stroke by CT or MRI [14]; (2) had first onset and course of disease in less than six months; (3) were right-handed and aged 35–80 years old; (4) were conscious and able to cooperate with the study; (5) meet the cognition assessment by Mini-Mental State Examination (MMSE) > 21; (6) signed the informed consent form. The exclusion criteria for this study were as follows: (1) with any MRI contraindications or other brain diseases; (2) with some severe comorbidities such as heart or renal function failure, pulmonary insufficiency, serious lung infection or liver dysfunction, and malignant tumor; (3) history of epilepsy or other neurological diseases and psychiatric disorders; (4) unable to complete the entire treatment and fear of acupuncture.

The eligible patients were randomized into either Group A or Group B through computer-generated randomization sequences. Opaque envelopes were used to hide the randomized data in this study. The group assignment was unknown for patients.

This study was based on the principles of the Declaration of Helsinki (Version Edinburgh 2000) [15] and obtained the approval of the Ethics Committee of the 1st Teaching Hospital of CDUTCM (No. 2011KL-002).

### 2.2. Acupuncture Interventions

Each subject of this study received basic standard treatments, including Anti-platelet aggregation therapy (100 mg aspirin once per day), neuroprotective treatment (500 mg citicoline per day), and other treatments according to the clinical symptoms.

In addition to standard treatment, the 11 patients in Group A received acupuncture treatment. The acupoints are as follows: Baihui (GV20), Fengchi (GB20), Quchi (LI11), Hegu (LI4), Yanglingquan (GB33), Zusanli (ST36), Sanyinjiao (SP6), and Xuanzhong (GB39). After needling, we used some Auxiliary techniques of acupuncture such as gentle manipulations of thrusting, lifting, and twirling to achieve de qi (including numbness, soreness, distention, heaviness, and other sensations), which is believed to be a crucial part of acupuncture efficacy. Participants were treated with 20 sessions in all, once per day, 5 consecutive sessions, and 2 days off in a week, with a duration of 30 min. The location of the acupoints is mainly determined by the national standard of the People's Republic of China (2006), Names and Locations of Acupoints (GB/T12346-2006). Moreover, Group B (10 patients) only received basic standard treatments.

### 2.3. Outcome Measurement

To assess clinical efficacy, modified Barthel Index (MBI), created by Shah et al. [16], has been used for symptom severity and quality of life among stroke patients. MBI is a daily life index that evaluates the ability of self-care independence, which consists of 10 items, including feeding, grooming, bathing, dressing, bowel and bladder care, using toilet, ambulation, transferring, and climbing stairs. Total score was from 0 to 100, with the higher score representing smaller nursing dependency [17]; on the contrary, the lower score indicated poor daily living ability. And it has obvious acceptability and similar psychometric characteristics for stroke patients during the rehabilitation process in hospital [18].

### 2.4. fMRI Scan

All brain images were obtained on the 3T Siemens MRI scanner (MAGNETOM Trio Tim, Siemens, Amberg, Germany) at the Huaxi MRI Center, West China Hospital of Sichuan University, China. The VBM protocol used a spin-echo planar image sequence with the following parameters: repetition time/echo time = 1,900 ms/2.26 ms, flip angle = 9°; in-plane matrix resolution = 256 × 256; slices = 176; field of view = 16 × 16 mm^2^; voxel size = 1 × 1 × 1 mm^3^. During the scan, each patient was blindfolded and their ears were plugged; moreover, in order to prevent the head from translating and rotating, all of them wore the foam cushions. Additionally, all female patients were scanned within one week after the menstrual cycle to avoid possible impact on brain activity and the size of the menstrual cycle [19, 20].

### 2.5. Statistical Analysis

#### 2.5.1. Clinical Variables

SPSS 19.0 software (SPSS, Chicago, IL, USA) was used to analyze the clinical variables by two blinded evaluators. The numerical variables comparisons within and between-group were performed using analysis of variance and the Kruskal-Wallis test. A two-sided test was applied for all available data. Categorical variables were calculated by a *X*^2^ test and described as *n* (percentage). Continuous variables were presented as the mean with 95% confidence intervals (CI). A *P* value < 0.05 was considered to be statistically significant.

#### 2.5.2. Voxel-Based Morphometry Analysis

Voxel-based morphometry (VBM) with Diffeomorphic Anatomical Registration using Exponentiated Lie Algebra (DARTEL) was conducted [21]. DARTEL has been shown to produce a more accurate registration than the standard VBM procedure and enables increased sensitivity to findings such as the correlation between grey matter volume and several measures such as age. After image acquisition by MRI, all T1-weighted MR images were analyzed using Statistical Parametric Mapping 8 (SPM8) (Wellcome Department of Cognitive Neurology, London, UK) in Matlab (Math Works, Natick, MA, USA). First, the “New Segmentation” algorithm from SPM8 was applied to every T1-weighted MR image to extract tissue maps corresponding to grey matter, white matter, and cerebrospinal fluid (CSF). This algorithm, which is an improvement on the unified segmentation algorithm [22], uses a Bayesian framework to iteratively perform the probabilistic tissue classification and spatial non-linear deformation in terms of Montreal Neurological Institute (MNI) space. Next, these segmented tissue maps were used to create a customized, more population-specific template using the DARTEL template-creation tool [21]. DARTEL estimates the best set of smooth deformations working from every subject's tissues to their common average, applies the deformations to create a new average, and then reiterates the process until convergence is achieved. We used a set of standard MNI tissues maps and a multivariate tissue-affinity-registration algorithm provided by SPM and DARTEL for that process. At the end of the process, each subject's grey matter map was warped using its corresponding smooth, reversible deformation parameters to transform it to the custom template space and then to the MNI standard space. Finally, the warped modulated grey matter images were smoothed by convolving an 8 × 8 × 8 mm^3^ full-width at half-maximum isotropic Gaussian kernel. After completing these image analyses, we obtained smoothed modulated grey matter images to be used for the statistical analysis. The significance of group differences was set at *P* < 0.05 using family-wise error correction.

#### 2.5.3. Correlation Analysis between Scales Score and GMD

The peak voxel and neighboring 100 voxels for each subject exhibiting GMV changes (*P* < 0.05, TFCE FWE correction) were selected as the region of interest (ROI). Then, we performed ROI-wise correlation analyses to evaluate whether the changes in GMV would be associated with the changes in the clinical variables (MBI scores). After correcting the volume, Pearson correlation coefficients were calculated between the mean volumes of the GMV and clinical variables (MBI scores).

## 3. Results

From January 2011 and December 2013, 21 ischemic stroke patients were recruited and randomly assigned in this study. In all, 21 patients all finished the treatment and fMRI scans, the lesion locations of which were primarily in the left basal ganglia. Demographic and clinical characteristics of 21 patients (11 in Group A and 10 in Group B) were shown in the Table 1. There were no significant differences in the demographics, including age, sex, and disease status as indicated by, for example, duration of symptoms, MBI score, and MMSE score, which did not differ between the two groups (*P* > 0.05).

**Figure 1:**
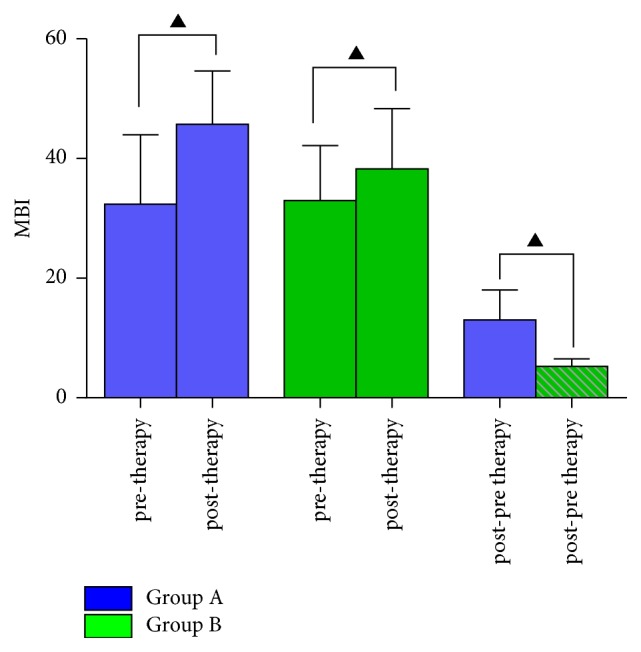
The changes of MBI performance with different intervention therapies. Paired *t*-test analyses showed significant increase of MBI scores from pre- to post-treatment in Group A and Group B. The change degree of MBI in patients with acupuncture treatment showed a significant enhancement comparing with the patients with conventional treatment (post-pre therapy in two groups). ^▲^*P* < 0.05.

After treatment intervention, a significant difference was found in MBI scores between two groups (Group A: from 32.4 ± 7.8 to 44.9 ± 6.4; Group B: from 33.00 ± 6.6 to 38.3 ± 7.2; *P* = 0.00). Additionally, the difference in change degree of MBI scores showed a significant improvement in the Group A, compared with Group B (Group A: 13.0 ± 3.58; Group B: 5.275 ± 0.902; *P* = 0.001) (Figure 1).

### 3.1. Changes in Grey Matter Volume after Treatment

**Figure 2:**
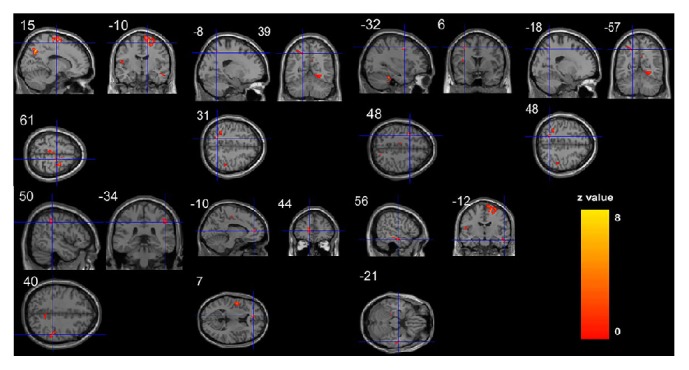
Cerebral GMV changes in ischemic stroke patients after acupuncture treatment. After acupuncture treatment in group A, ischemic stroke patients showed higher GMV in the left frontal lobe, precentral gyrus, Superior Parietal gyrus, Anterior Cingulate cortex, and Middle Temporal gyrus and lower GMV in the right frontal lobe and Inferior Parietal gyrus. before treatment versus after treatment in group A, *P* < 0.05; family-wise error corrected with a minimal cluster size of 30 voxel.

In Group A, an increase in the cerebral grey matter volume was observed after treatment in the left frontal gyrus, left precentral gyrus (BA6), left superior parietal gyrus, left anterior cingulate cortex (BA32), and left middle temporal gyrus. A decrease in the cerebral grey matter volume was detected in the right frontal gyrus, right inferior parietal gyrus, and right middle cingulate cortex (*P* < 0.05, family- wise error corrected with a minimal cluster size of 20 voxels) (Table 2) (Figure 2).

**Figure 3:**
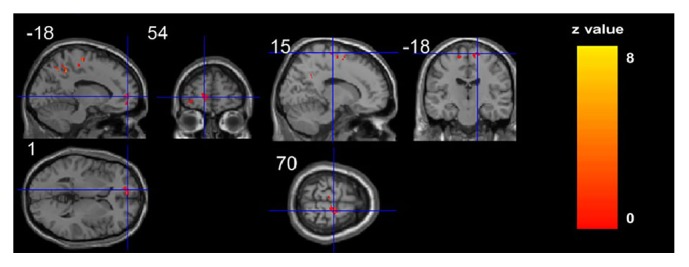
Cerebral GMV changes in ischemic stroke patients after conventional treatment. After conventional treatment in group B, ischemic stroke patients showed higher GMV in the right precentral gyrus (BA6) and inferior frontal gyrus (BA10). Before treatment versus after treatment in group B, *P* < 0.05; family-wise error corrected with a minimal cluster size of 30 voxels.

In Group B, an increase was observed after treatment in the right precentral gyrus (BA6), and right inferior frontal gyrus (BA10) (*P* < 0.05, family- wise error corrected with a minimal cluster size of 20 voxels) (Table 3) (Figure 3).

### 3.2. Correlations between GMV and Clinical Scale Scores

**Figure 4:**
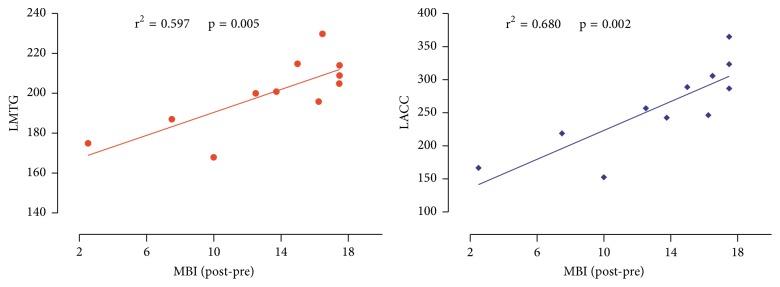
The correlation coefficients of brain grey structure changes and clinical variables. Note: LMTG: left middle temporal gyrus; LAGG: left anterior cingulate cortex; *r*: correlation coefficient; post-pre: posttreatment minus pretreatment.

In Group A, the increase in MBI score was significantly related to the GMV increase in the left middle temporal gyrus (*r*^2^ = 0.597, *P* = 0.005) and left anterior cingulate cortex (*r*^2^ = 0.680, *P* = 0.002) (Figure 4) (*P* < 0.01, corrected).

## 4. Discussion

Previous studies have demonstrated positive effects on functional recovery by using acupuncture in stroke patients [7, 23, 24]. Our present study also demonstrated acupuncture can improve the ability of daily life of stroke patients. Moreover, the novel key finding of the present neuroimaging study was that acupuncture can lead to structural reorganization in the recovering brain of stroke. Compared with patients who only received conventional treatment (Group B), the patients who received acupuncture treatment (Group A) showed extensive cerebral GMV changes in different regions. In Group A, the GMV was significantly increased in the left frontal lobe areas and left “default mode” network area, and the cerebral regions which GMV obviously decreased were found in the right frontal gyrus and right inferior parietal gyrus. In addition, the cerebral GMV changes in the Group B are right precentral gyrus and right inferior frontal gyrus. Moreover, we found the GMV changes in the left middle temporal gyrus (MTG) and left anterior cingulate cortex (ACC) positively are correlated with the behavioral changes of daily life. The different neuroplasty induced by acupuncture compared to conventional medicine indicates that acupuncture may enhance the role of conventional therapy. The grey matter (GM) tissue changes especially those involved in motor and cognition areas may be the potential therapy target and the potential mechanism of acupuncture.

### 4.1. Acupuncture Modulates the Motor Cortex Areas of Strokes

Numerous studies had demonstrated that both structural and functional reorganizations would occur in patiens following subcortical stroke, and the neuroplasticity changes in structural and functional levels were closely correlated to motor recovery of strokes [11, 25–27]. In the current study, the VBM analysis approach was used to the explore the effects of acupuncture on structural changes in the ischemic stroke patients. Our results showed significant alterations of grey matter structure in some motor-related regions, which probably interpret the mechanism of acupuncture on patients motor recovery from stroke. In the VBM analysis, there was a significant degeneration the contralesional frontal lobule; in contrast, the ipsilesional frontal lobule, precentral gyrus, and superior parietal gyrus increased obviously after four-week acupuncture treatments. The precentral gyrus plays a role in the relationship between motor function and the primary motor cortex. Additionally, the frontal and parietal lobe are also the key motor-related areas. The primary motor cortex (M1) and secondary motor areas (SMA) in the frontal and parietal lobe are recognised to the inter-regional corticocortical connectivity between key areas of the human motor network [28, 29]. Particularly, the premotor area of the frontal lobe and the supplementary motor area might act a potential substrate for brain reorganization after stroke as they have direct access to M1, as well as to the spinal cord [30]. Previous studies have reported that ipsilesional premotor areas such as ventral premotor cortex (PMv) and their interplay with M1 are contributing to motor function, spontaneous recovery [31], and also motor learning after stroke [32]. In monkeys, rehabilitation after stroke involves the primary motor cortex, as evidenced by changes in brain activity during recovery of hand function [33]. In addition, a recent fMRI research with multi-modality approach has identified the bi-hemispheric structural alterations after stroke and also reflected the increase in GMV of the contralesional SMA [25], suggesting that structural plasticity was associated with motor recovery. In our study, acupuncture treatments have induced the GMV decrease in contralesional hemisphere and GMV increase in the ipsilesional hemisphere. Another fMRI study with similar results considered that recovery of motor function after stroke is associated with normalization of activity in overactive brain regions [34]. Similar to the above study, the GMV of the patients in the group B mainly increased in the contralesional hemisphere and showed overactive in the present study. However, after receiving acupuncture treatment, the GMV in the contralesional frontal gyrus of strokers was induced to reduce. The result suggested that acupuncture might reduce the overactiveness of contralesional hemisphere. On the other hand, the GMV of ipsilesional motor-related area increased in the present study. Grefkes and Fink demonstrated the primary motor cortex of undamaged side in strokes had an interhemispheric inhibition on the injured side [35]. From this perspective, acupuncture might weaken the inhibitory effect by decreasing the GMV aggrandize of undamaged side, so the cerebral GMV of the damaged side increases, as a result of promoting the motor recovery.

### 4.2. Acupuncture Modulates the DMN of Strokers

Apart from the motor deficiency, emotion and cognition disorders, such as depression, confusion, and forgetfulness, are also the common complications of stroke patients and occur at a high incidence. For instance, recent studies have shown that at least 30–60% of post-stroke patients present symptoms of depression, which seriously restricts their rehabilitation [36]. The previous behavioral results showed that acupuncture improved the depression [37] or cognitive impairments [38] better than conventional therapy. In this study, acupuncture treatment elicited more extensive and remarkable cerebral structural changes as compared with conventional treatment. The left ACC and left MTG were only found in the acupuncture group and not in the conventional group (Tables 2 and 3 and Figures 2 and 3). The majority of these regions in the acupuncture group belong to “default mode” network (DMN).

The DMN is a brain network that presents as deactivated regions at rest, and various goal-directed [39], as well as emotional stimuli; this network can be activated. Our previous study indicated that, as compared with healthy subjects, stroke patients showed lower GMV in MTG [13]. In addition, Shi et al. [40] also found decreased grey matter volume in prefrontal cortex and cingulate cortex in stroke patients, which were also the key regions of DMN. The results suggested that successful treatment should modulate this network.

The ACC and MTG, considered to as key parts of the DMN [41, 42], play important roles in processing and modulating episodic memory [43], depression, and anxiety [44]. The ACC has been regarded as a core region involved in generating emotional responses, and its abnormal functioning has been linked to many psychiatric conditions [45], including memory and cognitive processing and their interactions with other brain networks related to conscious awareness [46]. The middle temporal gyrus was involved in episodic memory processing [43]. In some other fMRI studies it was found that, following a stroke, patients presented with delayed memory dysfunction and reduced functional connectivity in the temporal regions, prefrontal cortex, and cingulate gyrus within the DMN compared with healthy subjects [47]. Therefore, the structure changes of ACC and MTG areas might be the potential mechanism and therapy responses of acupuncture treatment for emotion and cognition recovery of strokes.

Interestingly, the current study found that the GMV increases of ACC and MTG are positively related to the increase in MBI score. This means that the GMV increases induced by acupuncture in these regions were associated with the improvement in ability of daily life. Nowadays, the cognitive impairments that might contribute to poor executive function have been documented before. For example, recent studies [48] found the function connection of ACC and MTG impaired of cirrhotic patients. They demonstrated that the function connection reduction within cognitive networks including DMN, executive control (ECN), and salience (SN) and performed significantly worse as reflected by the longer time with more errors to complete the Stroop task. So they thought slower psychomotor speed and impaired cognitive flexibility could consequently lead to executive dysfunction. Besides, relative to emotion and cognition function, some researchers found the ACC also had some relationship with the motor network. Treserras et al. [49] proved the posterior cingulate cortex (PCC) and ACC played an important role on interaction between DMN and sensorimotor network (SMN) during movement-readiness state. They claimed that the two networks were functionally correlated through an interaction between the PCC and ACC during movement-readiness but not functionally correlated during rest, and the ACC would have a motivational role or could generate predictions about the movement. Additionally, ACC was found to be implicated in attentional control, the execution or inhibition of motor commands [50]. Another study on bipolar disorder also suggested impaired ACC might modulate between emotion dysregulation and motor processing in youths with bipolar disorder [51]. In the present study, after 4 weeks of acupuncture treatment, the left ACC and MTG significantly increased and showed positive relationship to the increase in MBI score. The results were partly in line with those of a study by Zhang et al. [52], who found that acupuncture at Yanglingquan (GB34) improved the motor function by increasing DMN connectivity in the ACC and posterior cingulate cortex (PCC). The results suggested that, compared with conventional treatment, acupuncture treatment might improve the ability of daily life by not only affecting the motor regions but also modulating the ACC and MTG.

Hence, we speculated that the modulatory effects of acupuncture on the DMN of stroke patients might partly be explicated as the recovery of the cognitive ability and motor recovery.

## 5. Conclusions

In summary, our study showed that acupuncture can evoke pronounced structural reorganization. The frontal areas and the network of DMN of brain areas which related to motor and cognition recovery may be the potential therapy target and the potential mechanism of acupuncture treatment for ischemic stroke.

*Limitations*. The sample size of this study was small, and future investigations need a larger sample size for statistically accurate analysis. In addition, we found some significantly obvious changes in the cerebral areas related to cognition or emotion. There is no doubt that these changes could seriously restricts the rehabilitation of patients. However, more professional scales would be used to evaluate the emotion and cognition recovery, and then research the relationship between scales and brain changes.

## Figures and Tables

**Table 1:** Demographic and clinical characteristics of patients (*n* = 21).

Characteristic	Group A	Group B	*P* value
No. of patients (*n*)	11	10	-
No. of women, *n* (%)	4 (36.364)	5 (50.000)	0.497
Age (y), mean (95% CI)	69.364 (61.245, 77.483)	61.300 (53.362, 69.238)	0.127
Course of disease (D), mean (95% CI)	52.818 (22.348, 83.289)	52.200 (18.001, 86.399)	0.976
MBI score, mean (95% CI)	32.386 (24.613, 40.160)	32.980 (26.378, 39.572)	0.898
MMSE score, mean (95% CI)	21.000 (19.725, 22.275)	21.800 (20.262, 23.338)	0.380

*Notes*. Group A received standard conventional treatment and acupuncture treatment; Group B only received standard conventional treatment. CI: confidence interval; MBI: modified Barthel index; MMSE: Mini-Mental State Examination; *N*: number; %: percent; y: year; D: days.

**Table 2:** The cerebral GMV changes in Group A after treatment (end of treatment minus baseline).

Region	Side	Talairach			*t* value	BA	Voxel	Sign
		*X*	*Y*	*Z*				
frontal lobe	L	15	−10	61	7.00	-	1768	↑
	R	−8	39	31	6.24	-	674	↓
Precentral gyrus	L	−32	6	48	7.13	BA6	233	↑
Superior Parietal gyrus	L	−18	−57	48	6.98	-	358	↑
Inferior Parietal gyrus	R	50	−34	40	6.66	-	308	↓
Anterior Cingulate cortex	L	−10	44	7	6.44	BA32	378	↑
Middle Temporal gyrus	L	56	−12	−21	6.18	BA21-	230	↑

*Notes*. Group A received standard conventional treatment and acupuncture treatment. BA: Brodmann area; L: left; R: right; before treatment versus after treatment in group A, *P* < 0.05; family-wise error corrected with a minimal cluster size of 30 voxels.

**Table 3:** The cerebral GMV changes in Group B after treatment (end of treatment minus baseline).

Region	Side	Talairach			*t *value	BA	Voxel	Sign
		*X*	*Y*	*Z*				
precentral gyrus	R	15	−18	70	6.68	BA6	221	↑
Inferior frontal gyrus	R	−18	54	1	6.37	BA10	232	↑

## Data Availability

The data used to support the findings of this study are available from the corresponding author upon request.

## References

[1] Strong K., Mathers C., Bonita R. (2007). Preventing stroke: saving lives around the world. *The Lancet Neurology*.

[2] Liu M., Wu B., Wang W., Lee L., Zhang S., Kong L. (2007). Stroke in China: epidemiology, prevention, and management strategies. *The Lancet Neurology*.

[3] Feigin V. L., Forouzanfar M. H., Krishnamurthi R. (2014). Erratum: Global and regional burden of stroke during 1990-2010: Fi ndings from the Global Burden of Disease Study 2010 (Lancet (2014) 383 (245-255)). *The Lancet*.

[4] NIN Consensus Development Panel on Acupuncture (1998). NIH consensus conference. Acupuncture. *The Journal of the American Medical Association*.

[5] Salom-Moreno J., Sánchez-Mila Z., Ortega-Santiago R., Palacios-Ceña M., Truyol-Domínguez S., Fernández-De-Las-Peñas C. (2014). Changes in spasticity, widespread pressure pain sensitivity, and baropodometry after the application of dry needling in patients who have had a stroke: A randomized controlled trial. *Journal of Manipulative and Physiological Therapeutics*.

[6] Zhang S., Wu B., Liu M. (2015). Acupuncture efficacy on ischemic stroke recovery: multicenter randomized controlled trial in China. *Stroke*.

[7] Yang A., Wu H. M., Tang J., Xu L., Yang M., Liu G. J. (2016). Acupuncture for stroke rehabilitation. *Cochrane Database of Systematic Reviews*.

[8] Särkämö T., Ripollés P., Vepsäläinen H. (2014). Structural changes induced by daily music listening in the recovering brain after middle cerebral artery stroke: A voxel-based morphometry study. *Frontiers in Human Neuroscience*.

[9] Xing S., Lacey E. H., Skipper-Kallal L. M. (2016). Right hemisphere grey matter structure and language outcomes in chronic left hemisphere stroke. *Brain*.

[10] Krause T., Asseyer S., Taskin B. (2016). The Cortical Signature of Central Poststroke Pain: Gray Matter Decreases in Somatosensory, Insular, and Prefrontal Cortices. *Cerebral Cortex*.

[11] Miao P., Wang C., Li P. (2018). Altered gray matter volume, cerebral blood flow and functional connectivity in chronic stroke patients. *Neuroscience Letters*.

[12] Gauthier L. V., Taub E., Perkins C., Ortmann M., Mark V. W., Uswatte G. (2008). Remodeling the brain: plastic structural brain changes produced by different motor therapies after stroke. *Stroke*.

[13] Wu P., Zhou Y.-M., Zeng F. (2016). Regional brain structural abnormality in ischemic stroke patients: A voxel-based morphometry study. *Neural Regeneration Research*.

[14] Han Y., Lv H.-H., Liu X. (2015). Influence of Genetic Polymorphisms on Clopidogrel Response and Clinical Outcomes in Patients with Acute Ischemic Stroke CYP2C19 Genotype on Clopidogrel Response. *CNS Neuroscience & Therapeutics*.

[15] Salako S. E. (2006). The Declaration of Helsinki 2000: Ethical principles and the dignity of difference. *Medicine and Law*.

[16] Shah S., Vanclay F., Cooper B. (1989). Improving the sensitivity of the Barthel Index for stroke rehabilitation. *Journal of Clinical Epidemiology*.

[17] Quinn T. J., Langhorne P., Stott D. J. (2011). Barthel index for stroke trials: development, properties, and application. *Stroke*.

[18] Hsueh I.-P., Lin J.-H., Jeng J.-S., Hsieh C.-L. (2002). Comparison of the psychometric characteristics of the functional independence measure, 5 item Barthel index, and 10 item Barthel index in patients with stroke. *Journal of Neurology, Neurosurgery & Psychiatry*.

[19] Hagemann G., Ugur T., Schleussner E. (2011). Changes in brain size during the menstrual cycle. *PLoS ONE*.

[20] Veldhuijzen D. S., Keaser M. L., Traub D. S., Zhuo J., Gullapalli R. P., Greenspan J. D. (2013). The role of circulating sex hormones in menstrual cycle-dependent modulation of pain-related brain activation. *PAIN*.

[21] Ashburner J. (2007). A fast diffeomorphic image registration algorithm. *NeuroImage*.

[22] Ashburner J., Friston K. J. (2005). Unified segmentation. *NeuroImage*.

[23] Chen L., Fang J., Ma R. (2016). Additional effects of acupuncture on early comprehensive rehabilitation in patients with mild to moderate acute ischemic stroke: A multicenter randomized controlled trial. *BMC Complementary and Alternative Medicine*.

[24] Chen F., Qi Z., Luo Y. (2014). Non-pharmaceutical therapies for stroke: Mechanisms and clinical implications. *Progress in Neurobiology*.

[25] Fan F., Zhu C., Chen H. (2013). Dynamic brain structural changes after left hemisphere subcortical stroke. *Human Brain Mapping*.

[26] Cai J., Ji Q., Xin R. (2016). Contralesional cortical structural reorganization contributes to motor recovery after sub-cortical stroke: a longitudinal voxel-based morphometry study. *Frontiers in Human Neuroscience*.

[27] Nakashima A., Moriuchi T., Mitsunaga W. (2017). Prediction of prognosis of upper-extremity function following stroke-related paralysis using brain imaging. *Journal of Physical Therapy Science*.

[28] Schulz R., Park E., Lee J. (2017). Interactions Between the Corticospinal Tract and Premotor-Motor Pathways for Residual Motor Output After Stroke. *Stroke*.

[29] Carson R. G. (2005). Neural pathways mediating bilateral interactions between the upper limbs. *Brain Research Reviews*.

[30] Dum R. P., Strick P. L. (2002). Motor areas in the frontal lobe of the primate. *Physiology & Behavior*.

[31] Rehme A. K., Eickhoff S. B., Wang L. E., Fink G. R., Grefkes C. (2011). Dynamic causal modeling of cortical activity from the acute to the chronic stage after stroke. *NeuroImage*.

[32] Johansen-Berg H., Dawes H., Guy C., Smith S. M., Wade D. T., Matthews P. M. (2002). Correlation between motor improvements and altered fMRI activity after rehabilitative therapy. *Brain*.

[33] Nudo R. J., Milliken G. W., Jenkins W. M. (1996). Use-dependent alterations of movement representations in primary motor cortex of adult squirrel monkeys. *The Journal of Neuroscience*.

[34] Rehme A. K., Eickhoff S. B., Rottschy C., Fink G. R., Grefkes C. (2012). Activation likelihood estimation meta-analysis of motor-related neural activity after stroke. *NeuroImage*.

[35] Grefkes C., Fink G. R. (2014). Connectivity-based approaches in stroke and recovery of function. *The Lancet Neurology*.

[36] Schulte-Altedorneburg M., Bereczki D. (2014). Post-stroke depression. *Orvosi Hetilap*.

[37] Lu C.-Y., Huang H.-C., Chang H.-H. (2017). Acupuncture Therapy and Incidence of Depression after Stroke. *Stroke*.

[38] Huang J., You X., Liu W. (2017). Electroacupuncture ameliorating post-stroke cognitive impairments via inhibition of peri-infarct astroglial and microglial/macrophage P2 purinoceptors-mediated neuroinflammation and hyperplasia. *BMC Complementary and Alternative Medicine*.

[39] Raichle M. E., MacLeod A. M., Snyder A. Z., Powers W. J., Gusnard D. A., Shulman G. L. (2001). A default mode of brain function. *Proceedings of the National Acadamy of Sciences of the United States of America*.

[40] Shi Y., Zeng Y., Wu L. (2017). A Study of the Brain Abnormalities of Post-Stroke Depression in Frontal Lobe Lesion. *Scientific Reports*.

[41] Yoo S., Teh E., Blinder R. A., Jolesz F. A. (2004). Modulation of cerebellar activities by acupuncture stimulation: evidence from fMRI study. *NeuroImage*.

[42] Hui K. K. S., Liu J., Marina O. (2005). The integrated response of the human cerebro-cerebellar and limbic systems to acupuncture stimulation at ST 36 as evidenced by fMRI. *NeuroImage*.

[43] Tuladhar A. M., Snaphaan L., Shumskaya E. (2013). Default Mode Network Connectivity in Stroke Patients. *PLoS ONE*.

[44] Lassalle-Lagadec S., Sibon I., Dilharreguy B., Renou P., Fleury O., Allard M. (2012). Subacute default mode network dysfunction in the prediction of post-stroke depression severity. *Radiology*.

[45] Etkin A., Egner T., Kalisch R. (2011). Emotional processing in anterior cingulate and medial prefrontal cortex. *Trends in Cognitive Sciences*.

[46] Lavin C., Melis C., Mikulan E., Gelormini C., Huepe D., Ibañez A. (2013). The anterior cingulate cortex: an integrative hub for human socially-driven interactions. *Frontiers in Neuroscience*.

[47] Liu J., Wang Q., Liu F. (2017). Altered functional connectivity in patients with post-stroke memory impairment: A resting fMRI study. *Experimental and Therapeutic Medicine*.

[48] Yang Z., Chen H., Chen Q., Lin H. (2018). Disrupted Brain Intrinsic Networks and Executive Dysfunction in Cirrhotic Patients without Overt Hepatic Encephalopathy. *Frontiers in Neurology*.

[49] Treserras S., Boulanouar K., Conchou F. (2009). Transition from rest to movement: Brain correlates revealed by functional connectivity. *NeuroImage*.

[50] Benady-Chorney J., Yau Y., Zeighami Y., Bohbot V. D., West G. L. (2018). Habitual action video game players display increased cortical thickness in the dorsal anterior cingulate cortex. *NeuroReport*.

[51] King J. B., Anderson J. S., Yurgelun-Todd D. A., Subramaniam P., Ehrler M. R., Lopez-Larson M. P. Decreased anterior cingulate activation in a motor task in youths with bipolar disorder. *Journal of Child Psychology and Psychiatry*.

[52] Zhang Y., Li K., Ren Y. (2014). Acupuncture modulates the functional connectivity of the default mode network in stroke patients. *Evidence-Based Complementary and Alternative Medicine*.

